# Epidemiology of maxillofacial fractures in northwest China: an 11-year retrospective study of 2240 patients

**DOI:** 10.1186/s12903-023-03006-x

**Published:** 2023-05-23

**Authors:** Jingjing Mao, Xiaojie Li, Kun Cao, Jiawen Xue, Min Wang, Di Yan, Zhongwei Zhou

**Affiliations:** 1 Ningxia Medical University, No. 1160, Sheng Li South Road, Yinchuan, 750004 Ningxia P.R. China; 2 Department of Dental and Endodontic Diseases, General Hospital of Ningxia Medical University, No. 804, Sheng Li South Road, Yinchuan, 750004 Ningxia P.R. China; 3 Department of Oral and Maxillofacial Surgery, General Hospital of Ningxia Medical University, No. 804, Sheng Li South Road, Yinchuan, 750004 Ningxia P.R. China; 4 Department of Stomatology, The Eighth People’s Hospital of Jinan, No. 68, Xin Xing Road, Jinan, 271100 Shandong P.R. China; 5 Institute of Medical Sciences, General Hospital of Ningxia Medical University, No. 804, Sheng Li South Road, Yinchuan, 750004 Ningxia P.R. China

**Keywords:** Maxillofacial fracture, Retrospective study, Epidemiology, Aetiology

## Abstract

**Background:**

The aim of this study was to determine the epidemiological pattern of maxillofacial fractures in northwestern China by retrospectively analysing the demographics, aetiologies, concomitant injuries, fracture sites, and management.

**Methods:**

A 10-year retrospective analysis of 2240 patients with maxillofacial fractures admitted to the General Hospital of Ningxia Medical University was conducted. The extracted data included sex, age, aetiology, fracture site, concomitant injuries, time of treatment, therapeutic approaches and complications. Statistical analyses were performed, including descriptive analysis and the chi-square test. Logistic regression was used to determine the impact factors of maxillofacial fractures and concomitant injuries. P values < 0.05 were considered statistically significant.

**Results:**

The age of the included patients ranged from 1 to 85 years, and the mean age was 35.88 ± 15.69 years. The male-to-female ratio was 3.9:1. The most frequent aetiology of maxillofacial fractures was road traffic accidents (RTAs) (56.3%), and the most common fracture sites were the anterior wall of the maxillary sinus, arcus zygomaticus and mandibular body. A total of 1147 patients (51.2%) were affected by concomitant injuries, with craniocerebral injury being the most common. Logistic regression analyses revealed increased risks of mid-facial fractures in elderly individuals (odds ratio (OR) = 1.029, P < 0.001) and females (OR = 0.719, P = 0.005). Younger patients had a higher risk of mandibular fractures (OR = 0.973, P < 0.001). RTAs increased the risk for mid-facial fractures and high falls increased the risk for mandibular fractures.

**Conclusions:**

The maxillofacial fracture pattern is correlated with sex, age and aetiology. Patients were mainly young and middle-aged males, and the main cause of injury was RTAs, mostly causing compound fractures. Medical staff must be systematically educated to comprehensively examine patients with injuries resulting from RTAs. The management of patients with fractures requires thorough consideration of the patient’s age, aetiology, fracture site, and concomitant injuries.

**Supplementary Information:**

The online version contains supplementary material available at 10.1186/s12903-023-03006-x

## Background

The maxillofacial region is one of the most important parts of human aesthetics, and it plays a key role in ensuring normal feeding, chewing, breathing and craniocerebral preservation. As the most prominently located part of the body, the maxillofacial region is prone to fractures and related soft tissue injuries when exposed to external forces.

Maxillofacial fractures have been reported to be one of the most prevalent traumas worldwide [1]. Complex maxillofacial fractures and concomitant injuries increase the difficulty of treating fractures. If maxillofacial fractures are not treated appropriately and promptly, they can have a substantial damaging influence on patients’ physical and psychological health, as well as cause aesthetic dissatisfaction. Clarifying maxillofacial fracture patterns can aid in establishing efficient preventative and therapeutic modalities in the public health system. However, the epidemiological characteristics of maxillofacial fractures vary greatly depending on numerous aspects, including geographical location, culture, economy, and era [2].

Therefore, the aim of this study was to determine the composition, morbidity characteristics, and clinical epidemiological status of maxillofacial fractures in northwestern China by retrospectively analysing the clinical data of patients with maxillofacial fractures who were admitted to the General Hospital of Ningxia Medical University over an 11-year period from 2011 to 2021.

## Patients and methods

### Study design and population

The study protocol was approved by the Medical Research Ethics Review Committee of the General Hospital of Ningxia Medical University (number KYLL-2022-0096). Informed consent was obtained from the study participants. All patients included in the study signed an informed consent form at the time of hospital admission and agreed to the use of their anonymized medical data for scientific research purposes. In the case of patients under the age of 18 years, the informed consent form was signed by a parent or legal guardian.

This was a retrospective study based on electronic medical records. The study participants were patients with maxillofacial fractures who were admitted to the General Hospital of Ningxia Medical University between January 2011 and December 2021.

The inclusion criteria were as follows: patients with maxillofacial fractures with complete case information. The exclusion criteria were as follows: patients with incomplete medical records; those with soft tissue injuries only; those with isolated nasal fractures; those with pathological fractures; and those with a history of maxillofacial fractures whose reason for visiting during this study was related to their past trauma history.

### Data collection

All data for this study were extracted from the hospital’s electronic medical record system. The following data were collected: sex, age, time and cause of injury, fracture site, concomitant injuries, time of treatment, treatment modalities, and complications. The included patients were identified by International Classification of Diseases (ICD) codes and subclassified by imaging presentation. To prevent bias, all observation records were checked twice by the authors who collected the data.

### Ethical considerations

Ethical considerations were considered throughout the study, and the patients’ names and medical information were kept completely confidential. No information or images could lead to identification of the study subjects. The participants’ medical histories were used solely for the purposes of this study.

### Statistical analysis

IBM SPSS Statistics version 22.0 was used to conduct statistical analysis. Descriptive statistics were calculated, and the chi-square test was used to analyse the collected data. Logistic regression was used to determine the impact of sex, age and different aetiologies of maxillofacial fractures and concomitant injuries. P values < 0.05 were considered statistically significant.

## Results

A total of 6737 patients with maxillofacial trauma were admitted to the hospital between January 1, 2011, and December 31, 2021. 2240 patients were ultimately included; 4497 patients were excluded due to having incomplete records, soft tissue injuries only, isolated nasal fractures and pathological fractures. Patients with a history of maxillofacial fractures whose reason for visiting during this study was related to their past trauma history were also excluded.

### Sex and age distribution

**Fig. 1:**
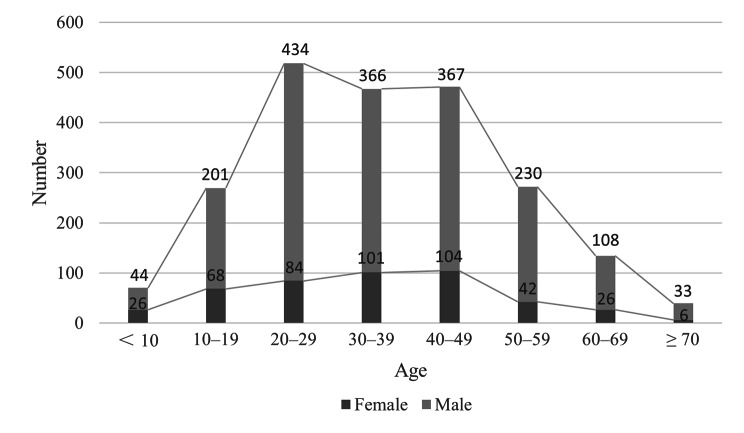
Age and sex distribution of patients

Of the 2240 patients included in this study, 1783 were male and 457 were female, with a male-to-female ratio of 3.9:1. The age of patients at the time of injury ranged from 1 to 85 years, with a median of 35 years and a mean of 35.88 ± 15.69 years. Moreover, 65.0% of the patients were aged from 20 to 49 years, including 1167 males and 289 females. Fractures least commonly occurred in the ≥ 70 year age group (Fig. 1). We observed statistically significant differences in the incidence rates by sex and age (χ^2^ = 27.67, P < 0.001).

### Aetiology of maxillofacial fractures

**Fig. 2:**
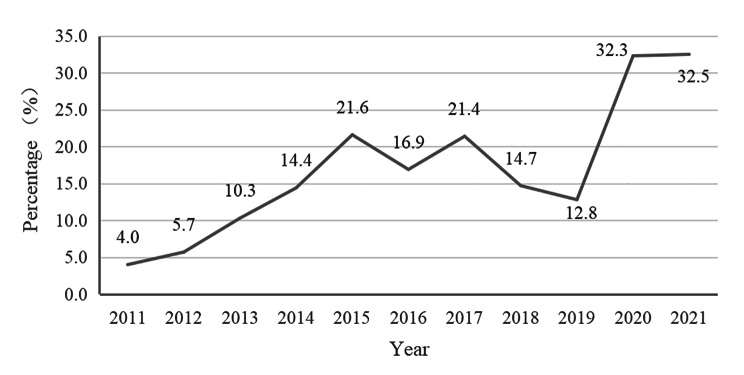
Proportion of electric-powered bike injuries due to RTAs according to year

In this study, road traffic accidents (RTAs) were the leading cause of injuries, followed by falls, being struck by objects, and high falls. Among the 1262 patients with maxillofacial fractures caused by RTAs, 926 (73.3%) were injured by vehicles (including motor vehicles and electric vehicles), followed by electric-powered bikes (18.5%) and bicycles (8.2%). Overall, the number of maxillofacial fractures caused by electric-powered bikes has been increasing recently (Fig. 2).

RTAs were the leading cause of injury in different age groups. Most of the patients who were injured by assault were between 20 and 39 years of age (78.7%). Elderly individuals (≥ 50 years of age) and children (< 10 years of age) had a significantly higher incidence of injuries caused by falls (Suppl. 1). Although the main cause of maxillofacial fractures was RTAs in both males and females, the rate of RTA-induced injuries was significantly higher in females than in males. However, the incidence of injuries caused by being struck by objects was lower in females than in males (Suppl. 2). Of the 286 patients who were struck by objects, 239 (83.6%) had work accidents, and 47 (16.4%) had sports accidents. Regarding fracture severity, fractures caused by assault, bicycles and falls were less severe, while those caused by vehicles, electric-powered bikes and high falls were more severe (Table 1).

**Table 1:** Distribution of maxillofacial fracture severity according to aetiology

Aetiology	Fracture severity			Total (%)
	One fracture (%)	Two fractures (%)	Multiple (≥ 3) fractures (%)	
Vehicle	209 (22.6)	225 (24.3)	492 (53.1)	926 (100)
Electric-powered bike	48 (20.6)	58 (24.9)	127 (54.5)	233 (100)
Bicycle	44 (42.7)	26 (25.3)	33 (32.0)	103 (100)
Assault	55 (45.1)	44 (36.1)	23 (18.8)	122 (100)
High fall	47 (24.4)	44 (22.8)	102 (52.8)	193 (100)
Fall	133 (40.3)	107 (32.4)	90 (27.3)	330(100)
Struck by objects	100 (35.0)	73 (25.5)	113 (39.5)	286 (100)
Explosion	1 (11.1)	2 (22.2)	6 (66.6)	9 (100)
Other	17 (44.7)	7 (18.4)	14 (36.9)	38 (100)
Total	654 (29.2)	586 (26.2)	1000 (44.6)	2240 (100)

### Concomitant injuries

A total of 1147 patients (51.2%) had 1991 associated injuries, with craniocerebral injuries (n=783, 39.3%) being the most common, followed by limb (n = 438, 22.0%), thoracic (n = 379, 19.0%), and oculus injuries (n = 189, 9.5%). Concomitant injuries to the spine (n = 103, 5.2%), abdomen (n = 44, 2.0%), and neck (n = 27, 1.4%) were relatively rare. Of the 1147 patients with concomitant injuries, 73.8% had fractures in two or more sites.

### Distribution of maxillofacial fractures

A total of 6645 fractures occurred in 2240 patients, with 2.97 fractures per patient. Most patients (n = 1586, 70.8%) had two or more fractures. Mid-facial fractures occurred mostly in the zygomatic region, including the zygomatic body, arcus zygomaticus, and lateral orbital wall, with a total of 2085 (43.2%) fractures. Additionally, maxillary fractures were the second most common fracture type after zygomatic fractures. There were 1059 fractures in the anterior wall of the maxillary sinus, which was the leading anatomical site of maxillary fractures, accounting for 55.4% (Suppl. 3). The mandibular body represented the primary mandibular fracture site, with 866 fractures observed, accounting for 47.5% of the general mandibular fractures. This was followed by condylar fractures (26.3%) and angle fractures (10.6%). Coronoid fractures were the least common, accounting for only 1.9% of all mandibular fractures (Suppl. 4).

### Treatment and complications

Of the 2240 patients, 1387 (61.9%) underwent surgery i.e., open reduction and internal fixation (ORIF), and 618 (27.6%) received conservative treatment, i.e., soft diet, intermaxillary fixation, closed reduction and intermaxillary traction combined with occlusal pads. A total of 235 patients (10.5%) received no treatment, including 207 with no intervention indications, i.e., dysfunction or facial deformity, 22 with medical cost concerns and 6 with psychological reasons. Most patients who received no treatment were elderly and had zygoma fractures (body of the zygoma bone and arcus zygomaticus).

Of the 2005 patients treated, 1322 (65.9%) were treated within 2 weeks of injury, 474 (23.6%) were treated within 2–4 weeks of injury, and 209 (10.4%) were treated more than 4 weeks after injury. Of these patients, 56 underwent ORIF within 48 h of injury, usually for open injuries. The majority (65.4%) of patients treated ≥ 2 weeks postinjury had concomitant injuries.

Temporary local infection was the leading postoperative complication, occurring in 66 patients (4.8%), and was well controlled by local open drainage of the operative area and systematic antibiotic application. Soft tissue pain and swelling were the leading complications in patients who received conservative treatment, followed by limitation of mouth opening, occlusal disorder and mandibular deviation. Facial asymmetry and introcession deformity were the leading complications among the patients who did not receive treatment due to medical costs and psychological reasons.

### Multifactor analysis by logistic regression

The risk of mid-facial fractures (p < 0.001) increased by 2.9% per year, but that of mandibular fractures (p < 0.001) decreased by 2.7% per year. Females were at higher risk of mid-facial fractures (by 28.1%) than males (p = 0.005), but there was no significant sex difference in the risk of mandibular fractures (p = 0.686). The risk of mid-facial fractures due to RTAs increased by 84.6% (p < 0.001) and that of mandibular fractures decreased by 17.7% (p = 0.030). Both high falls and being struck by an object increased the risk of mandibular fractures (by 84.6% and 32.1%, respectively) (Tables 2 and 3).

**Table 2:** Multivariate analysis of mid-facial fractures by logistic regression

	Mid-facial fracture		Crude odds ratio	Adjusted odds ratio	Adjusted 95% CI	Adjusted significance
	Yes	No				
	(n = 1487)	(n = 753)				
Age	38.12 ± 15.52	31.47 ± 15.09	1.029	1.029	1.023–1.035	<0.001
Sex						
Female	285	172	0.801	0.719	0.572–0.904	0.005
Male	1202	581				
Aetiology						
RTA	904	358	1.773	1.846	1.532–2.223	<0.001
Assault	62	60	0.507	0.527	0.363–0.765	0.001
High fall	117	76	0.769	0.825	0.605–1.125	0.224
Fall	196	134	0.709	0.711	0.555–0.910	0.007
Struck by an object	172	114	0.741	0.650	0.499–0.847	0.001

RTA, road traffic accident.

**Table 3:** Multivariate analysis of mandibular fractures by logistic regression

	Mandibular fracture		Crude odds ratio	Adjusted odds ratio	Adjusted 95% CI	Adjusted significance
	Yes	No				
	(n = 1147)	(n = 1093)				
Age	32.83 ± 15.04	39.22 ± 15.72	0.974	0.973	0.968–0.979	<0.001
Sex						
Female	230	227	0.911	0.956	0.768–1.190	0.686
Male	939	844				
Aetiology						
RTA	632	630	0.796	0.823	0.691–0.982	0.030
Assault	63	59	0.965	0.885	0.610–1.283	0.519
High fall	130	63	1.979	1.846	1.342–2.541	<0.001
Fall	161	169	0.840	0.819	0.644–1.043	0.105
Struck by an object	165	121	1.274	1.321	1.020–1.712	0.035

RTA, road traffic accident.

Furthermore, females with maxillofacial fractures were at higher risk of concomitant injuries (31.1%; p = 0.001). Elderly individuals were at greater risk of concomitant injuries (1.8% per year; p < 0.001). Patients with injuries caused by RTAs and high falls were at higher risk of concomitant injuries (152.6% and 35.8%, respectively; Table 4).

**Table 4:** Multivariate analysis of concomitant injuries by logistic regression

	Concomitant injuries		Crude odds ratio	Adjusted odds ratio	Adjusted 95% CI	Adjusted significance
	Yes	No				
	(n = 1147)	(n = 1093)				
Age	37.95 ± 16.16	33.72 ± 14.89	1.018	1.018	1.012–1.024	<0.001
Sex						
Female	210	247	0.768	0.689	0.552–0.861	0.001
Male	937	846				
Aetiology						
RTA	763	499	2.393	2.526	2.113–3.020	<0.001
Assault	25	97	0.227	0.233	0.148–0.365	<0.001
High fall	110	83	1.284	1.358	1.004–1.837	0.047
Fall	128	202	0.550	0.543	0.426–0.693	<0.001
Struck by an object	100	186	0.462	0.410	0.315–0.535	<0.001

RTA, road traffic accident.

## Discussion

Maxillofacial trauma is a significant contributor to systemic trauma, accounting for 17–32% of systemic trauma patients [3, 4]. According to the Global Burden of Disease, the global incidence of maxillofacial fractures is increasing [5], and domestic studies have revealed the same tendency [6, 7]. Determining the epidemiological pattern of maxillofacial trauma is of great importance to public health.

In the current study, the ratio of males to females with maxillofacial trauma in Northwest China was 3.9:1, which is similar to that reported worldwide [8] and in accordance with studies in Western [9], Southeast [10] and Northern China [11]. Such a sex difference is probably due to the higher incidence of outdoor activities conducted by males. The male-to-female ratio varies by region and period. Less developed regions have a larger male-to-female ratio than developed regions [6, 10, 12]. In Xinjiang, for example, the male-to-female ratio was as high as 4.9:1 [6], while it was merely 1.8:1 in Beijing [12]. For the same region, the male-to-female ratio has decreased in the last few years [6, 9, 11, 13]. This disparity could be attributed to the level of economic development. As the economy develops, females become more involved in productive social activities and are more prone to maxillofacial fractures as their socioeconomic status rises. In this study, the 20–49 year age group accounted for the most injuries (n = 1456, 65.0%), which is consistent with previous reports. People this age group are the main mass of social production activities and often participate in outdoor activities, which is associated with a higher risk of maxillofacial fractures [10].

Several studies have revealed that violence is the leading cause of maxillofacial fractures in developed countries [2, 8], while RTAs are the dominant cause in developing countries [2, 9]. Additionally, violence has become the major cause of injury in some low-income underdeveloped countries [14]. In this study, RTAs were the most common cause of maxillofacial fractures, with an incidence of 56.3%, which is higher than that reported in previous studies in the same area (41.9%) [13]. Therefore, it is important to emphasize and provide prevention methods for maxillofacial fractures caused by RTAs. Moreover, the proportion of RTAs related to electric-powered bikes is increasing annually, which reveals that methods such as developing robust traffic laws and regulations, increasing supervision and law enforcement, enhancing residents’ safety awareness through community publicity of traffic rules and encouraging of the use of helmets [15] need to be utilized to radically reduce the occurrence of traffic accident injuries. In this survey, the 20–49 year age group had the highest number of fractures caused by assault, whereas the 15–24 year age group had the highest number in prior reports [16]. This can be related to global population ageing [17], as well as cultural, social, and economic differences among countries.

Workplace accidents are becoming more common as society rapidly industrializes. In this study, work accidents accounted for 83.6% of fractures due to being struck by objects and 10.7% of the total 2240 patients, which is significantly higher than in developed countries [18, 19]. The male-to-female ratio the work accident group was 30.8:1, which is in line with earlier studies [20, 21], as male workers are more frequently engaged in physical and risky work than female workers. The misalignment of development and safety protection, that is, the emphasis on industrial development rather than injury prevention, is a major factor in the high and frequent incidence of work accidents.

The leading sites of maxillofacial fractures were the anterior wall of the maxillary sinus and the arcus zygomaticus. For mandible fractures, the most common sites were the mandibular body, condyle, and angle regions, which is consistent with findings worldwide [8, 22]. The incidence of maxillary fractures in this study was slightly higher than that in domestic studies [23], which may be attributed to the inclusion of all orbital walls in the maxillary statistics in this study. The incidence of maxillofacial fractures with concomitant injuries ranged from 18.0 to 47.7% [9, 19, 24], with craniocerebral and limb injuries having the highest incidences. In this study, 51.2% of the patients had concomitant injuries, which were related to the cause of injury, the force subjected, and the type of fracture. The presence of concomitant injuries changes the treatment plan and time for patients with maxillofacial fractures, making it difficult to recover their facial shape and function after injury. Therefore, a timely and correct assessment of a maxillofacial trauma patient’s overall condition and the presence of comorbid injuries at admission is crucial to their treatment and prognosis.

Sex, age and aetiology were strongly linked with fracture sites and the existence of concomitant injuries. Age has been reported as a protective factor for maxillofacial fractures [25], while some studies have presented the opposite conclusion [13, 19]. The effects of age on mandibular and mid-facial fractures were examined independently in this study. The findings revealed that younger patients were more likely to suffer from mandibular fractures, while the inverse was true for mid-facial fractures. This could be linked to maxillofacial anatomy. The younger a patient is (e.g., child), the less mature the maxilla, the more anterior the mandible, and the more vulnerable to external blows. In contrast, the older a patient is, the more developed and anterior the maxilla, and the greater the risk of fracture [26]. The finding that patients who experienced RTAs had the highest risk of mid-facial fractures shows that the mid-facial region is more vulnerable in RTAs. Females are more likely than males to sustain mid-facial fractures, which may be related to the fact that females in this study were more likely to have experienced RTAs. A study further analysed the impact of various means of transportation on maxillofacial fractures. The study showed that the risk of maxillofacial fractures from motor vehicle accidents increased by 220% when compared to other means of transportation accidents [27]. Therefore, motor vehicle accident injuries must be prevented by reinforcing vehicle restraints such as seat belt use, speed limits, and stricter drunk driving laws. RTA patients were more likely to sustain concomitant injuries, with motor vehicle accidents increasing the likelihood of craniocerebral injury by 520% when compared to other causes [28]. Injuries to key organs might be fatal if not treated immediately. Therefore, maxillofacial surgeons must pay special attention to patients who have sustained RTA injuries during the initial evaluation. Careful screening for concomitant injuries and vital signs before treating fractures is critical to prevent death from concomitant injuries.

The treatment of maxillofacial fractures includes conservative and surgical treatment [29]. Maxillofacial surgeons should determine treatment based on the site and severity of injury, concomitant injuries and patient age. ORIF is the preferred treatment option for maxillofacial fractures to restore the normal maxillofacial anatomical form and occlusal-dental relationship in patients [30–32]. Conservative treatment is mostly seen in children and adolescents with isolated condylar fractures [33], usually associated with force on the mandibular symphysis after falling that is transmitted to the condylar neck [34].

The General Hospital of Ningxia Medical University serves a population of approximately 20 million people, involving individuals from Ningxia and adjacent provinces. The maxillofacial fracture pattern in this hospital can basically represent the pattern in northwestern China. Therefore, this study can provide an important reference regarding the epidemiology of maxillofacial fractures in northwestern China. These data can be used by healthcare institutions to improve the examination, treatment, and care system for patients with maxillofacial fractures. The Centers for Disease Control and Prevention can also develop targeted education and prevention measures based on these data, which will effectively reduce the public health burden.

However, some limitations must be taken into consideration. The study only summarized the epidemiological results of one medical centre, and a multicentre observation could provide a more reliable reference. Soft tissue injuries are common in patients with maxillofacial trauma and including them in the statistics would provide more complete information. This was a retrospective study, so some information in the medical records may be incomplete or erroneous. The possibility of patients concealing facts and misreporting the cause of trauma cannot be ruled out.

## Conclusions

The age range in which people are prone to maxillofacial fractures in our region is 20–49 years old, with a male predominance. RTAs were the primary cause of injury. The majority of fractures occurred in the arcus zygomaticus of the zygoma, the anterior wall of the maxillary sinus, and the body and condyle of the mandible. More than half of the patients presented with a mix of other systemic injuries, the most prevalent of which was craniocerebral damage. Developing targeted preventive measures, especially strengthening education on traffic regulations and raising awareness of traffic safety, is essential to reduce the occurrence of maxillofacial fractures. There is a need to educate medical staff on the epidemiology of maxillofacial fractures to accelerate diagnosis and treatment.

## Electronic supplementary material

Below is the link to the electronic supplementary material.

Supplementary Material 1

Supplementary Material 2

Supplementary Material 3

Supplementary Material 4

## Data Availability

The data that support the findings of this study are available from the corresponding author upon request.
