# Supplementary figures and images for: Epidemiology of maxillofacial fractures in northwest China: an 11-year retrospective study of 2240 patients
Source: BMC Oral Health. 2023 May 23;23:313. doi: 10.1186/s12903-023-03006-x (PMC10204232; doi:10.1186/s12903-023-03006-x)

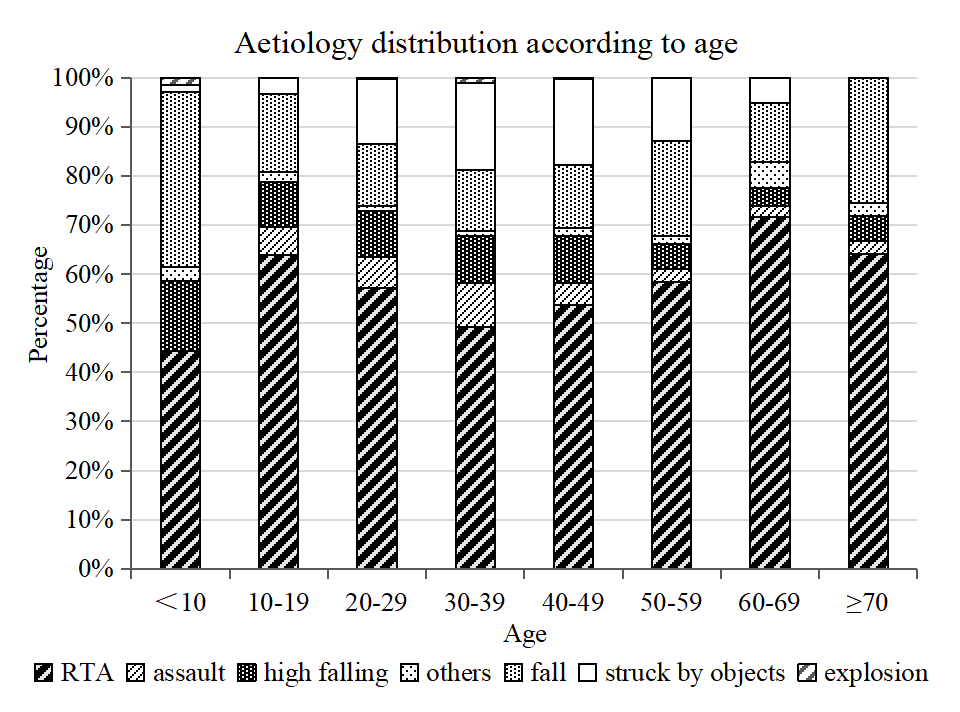

Supplement: Supplementary file 1 — Supplementary Material 1 [file 12903_2023_3006_MOESM1_ESM.bmp]

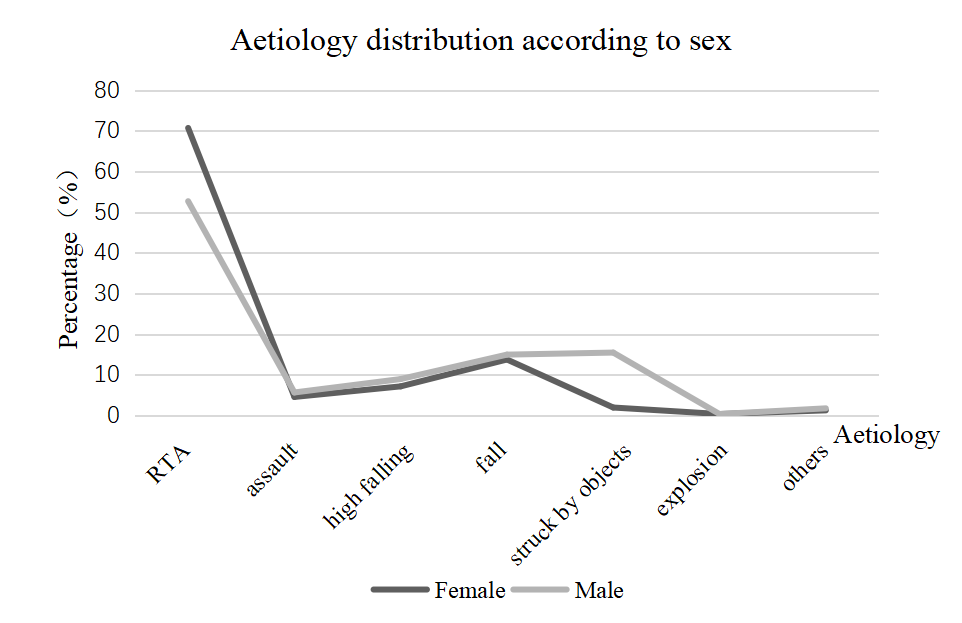

Supplement: Supplementary file 2 — Supplementary Material 2 [file 12903_2023_3006_MOESM2_ESM.bmp]
