# Supplementary material for: Epidemiology of maxillofacial fractures in northwest China: an 11-year retrospective study of 2240 patients
Source: BMC Oral Health. 2023 May 23;23:313. doi: 10.1186/s12903-023-03006-x (PMC10204232; doi:10.1186/s12903-023-03006-x)
Supplement: Supplementary file 3 — Supplementary Material 3 [file 12903_2023_3006_MOESM3_ESM.pdf]

Suppl. 3 Site distribution of mid-facial fractures

Anatomical location	Number	Percentage (%)
Zygoma		
Arcus zygomaticus	1009	20.9
Lateral orbital wall	685	14.2
Body of zygoma	391	8.1
Maxilla		
Anterior wall of maxillary sinus	1059	22.0
Lateral wall of maxillary sinus	295	6.1
Orbital floor	214	4.4
Alveolar crest	192	4.0
Palatine process	70	1.5
Pterygoid process	53	1.1
Frontal process	29	0.6
Nasal		
Nasal bone	504	10.5
Nasal septum	97	2.0
Cribriform plate of ethmoid	217	4.5
Vomer	6	0.1
Total	4821	100
