# Supplementary material for: Epidemiology of maxillofacial fractures in northwest China: an 11-year retrospective study of 2240 patients
Source: BMC Oral Health. 2023 May 23;23:313. doi: 10.1186/s12903-023-03006-x (PMC10204232; doi:10.1186/s12903-023-03006-x)
Supplement: Supplementary file 4 — Supplementary Material 4 [file 12903_2023_3006_MOESM4_ESM.pdf]

Suppl. 4 Site distribution of mandibular fractures

Anatomical location	Number	Percentage (%)
Body	866	47.5
Condyle	479	26.3
Angle	194	10.6
Ramus	91	5.0
Symphyseal region	83	4.5
Alveolar crest	77	4.2
Coronoid	34	1.9
Total	1824	100
